# Long-Term Consumption of Anthocyanin-Rich Fruit Juice: Impact on Gut Microbiota and Antioxidant Markers in Lymphocytes of Healthy Males

**DOI:** 10.3390/antiox10010027

**Published:** 2020-12-29

**Authors:** Isabel Anna Maria Groh, Alessandra Riva, Dominik Braun, Heidi G. Sutherland, Owen Williams, Tamara Bakuradze, Gudrun Pahlke, Elke Richling, Larisa M. Haupt, Lyn R. Griffiths, David Berry, Doris Marko

**Affiliations:** 1 Department of Food Chemistry and Toxicology, Faculty of Chemistry, University of Vienna, Waehringerstrasse 38, 1090 Vienna, Austria; isabelgroh@gmx.de (I.A.M.G.); dominik.braun@univie.ac.at (D.B.); gudrun.pahlke@univie.ac.at (G.P.); 2 Department of Experimental and Clinical Pharmacology and Pharmacogenomic, Division of Pharmacogenomic, University Hospital of Tuebingen, Wilhelmstrasse 56, 72074 Tuebingen, Germany; 3 Centre for Microbiology and Environmental Systems Science, Department of Microbiology and Ecosystem Science, University of Vienna, Althanstrasse 14, 1090 Vienna, Austria; alessandra.riva@univie.ac.at (A.R.); williams@microbial-ecology.net (O.W.); david.berry@univie.ac.at (D.B.); 4 Centre for Genomics and Personalised Health, Genomics Research Centre, School of Biomedical Sciences, Institute of Health and Biomedical Innovation University of Technology (QUT), Queensland, 60 Musk Ave., Kelvin Grove, QLD 4059, Australia; heidi.sutherland@qut.edu.au (H.G.S.); larisa.haupt@qut.edu.au (L.M.H.); lyn.griffiths@qut.edu.au (L.R.G.); 5 Food Chemistry and Toxicology, Department of Chemistry, University of Kaiserslautern, Erwin-Schroedinger-Strasse 52, D-67663 Kaiserslautern, Germany; bakurad@rhrk.uni-kl.de (T.B.); Richling@chemie.uni-kl.de (E.R.)

**Keywords:** anthocyanin, intervention study, nuclear factor erythroid 2 (NFE2)-related factor 2 (Nrf2), antioxidant effects, microbiota

## Abstract

Polyphenols are considered protective against diseases associated with oxidative stress. Short-term intake of an anthocyanin-rich fruit juice resulted in significantly reduced deoxyribonucleic acid (DNA) strand-breaks in peripheral blood lymphocytes (PBLs) and affected antioxidant markers in healthy volunteers. Consequently, effects of long-term consumption of fruit juice are of particular interest. In focus was the impact on nuclear factor erythroid 2 (NFE2)-related factor 2 (Nrf2), the Nrf2-regulated genes NAD(P)H quinone oxidoreductase 1 (*NQO-1*) and heme oxygenase 1 (*HO-1*) as well as effects on the gut microbiota. In a nine-week placebo-controlled intervention trial with 57 healthy male volunteers, consumption of anthocyanin-rich juice significantly increased *NQO-1* and *HO-1* transcript levels in PBLs compared to a placebo beverage as measured by real-time polymerase chain reaction (PCR). Three Nrf2-promotor single nucleotide polymorphisms (SNPs), analyzed by pyrosequencing, indicated an association between individual Nrf2 transcript levels and genotype. Moreover, the Nrf2 genotype appeared to correlate with the presence of specific microbial organisms identified by 16S-PCR and classified as *Spirochaetaceae*. Furthermore, the microbial community was significantly affected by the duration of juice consumption and intake of juice itself. Taken together, long-term consumption of anthocyanin-rich fruit juice affected Nrf2-dependent transcription in PBLs, indicating systemic effects. Individual Nrf2 genotypes may influence the antioxidant response, thus requiring consideration in future intervention studies focusing on the Nrf2 pathway. Anthocyanin-rich fruit juice had an extensive impact on the gut microbiota.

## 1. Introduction

Anthocyanins are polyphenols naturally abundant in the human diet and responsible for the red and purple color in vegetables and fruits such as grapes, bilberries and cherries [1]. The consumption of wild blueberries, a food source high in vitro antioxidant properties, has been associated with a diet-induced increase in ex vivo serum antioxidant status [2]. Epidemiological studies and associated meta-analyses strongly suggest that long-term consumption of diets rich in plant polyphenols offer protection against development of cancers, cardiovascular diseases, diabetes, osteoporosis and neurodegenerative diseases [3,4,5]. These diseases are often associated with oxidative stress, which is generated by an imbalance between the production of reactive oxygen species (ROS) and the antioxidant efficiency of the cells. However, cellular defense mechanisms, e.g., in the form of antioxidant enzymes such as superoxide dismutase, catalase and glutathione peroxidase, protect from major damage [6]. The nuclear factor erythroid 2 (NFE2)-related factor 2 (Nrf2)-signaling pathway represents one of the main cell defense mechanisms and is a major regulator of cell survival. The transcription factor Nrf2 controls the expression of genes essential for antioxidant defense and phase-II detoxification [7,8,9,10]. Under quiescent conditions Nrf2 is bound to Kelch-like ECH-associated protein 1 (Keap1) in the cytosol and is in homeostasis by basal biosynthesis and proteasomal degradation. In its active form, Nrf2 translocates into the nucleus and binds the antioxidant response element (ARE), thereby triggering the transcription of a wide variety of genes to protect the organism against xenobiotics and/or oxidative stress. AREs are located in the promoter region of several cell defense genes, comprising among others several phase-II detoxifying enzymes (glutathione-S-transferases (GSTs), γ-glutamyl-cysteine-ligase, etc.) and enzymes involved in antioxidant defense such as heme oxygenase 1 (HO-1) or NAD(P)H quinone oxidoreductase 1 (NQO-1). Consequently, increased expression of such protective enzymes in the cell generates a state of improved defense against oxidative stress [11].

Mutations within Keap1 and Nrf2 as well as the antioxidant phase-II enzymes involved in their interaction might lead to chronic activation or inactivation of the Nrf2 pathway. Alterations such as single nucleotide polymorphisms (SNPs) can affect antioxidant defense, as has been shown for Nrf2 [12]. A reduction of oxidative deoxyribonucleic acid (DNA) damage and an increase in glutathione (GSH) status was observed in healthy male volunteers after daily intake of 700 mL red multi-fruit juice over a four-week period [6]. However, the mode of action is still unclear. In a further human intervention study with healthy subjects and ileostomy volunteers lacking a colon, limitation of antioxidant and DNA-protective effects in comparison to healthy subjects suggested a role for colonic processes in bioactivity, supported by Nrf2-activating properties of the intestinal anthocyanin degradation product phloroglucinol aldehyde (PGA) [13]. These results indicate that digestive processes regulated by the intestinal microbiota may be very important for the bioactivity of anthocyanins. Another in vivo study with cranberry extract exerted beneficial metabolic effects of a high fat/high sucrose diet in wild-type mice, used to induce features of the metabolic syndrome found to be associated with a proportional increase in *Akkermansia* spp. in the intestinal microbiota [14]. Currently, approximately 500–1000 different microbial species inhabit the gastrointestinal tract, reaching the highest concentrations in the colon. The human gut harbors a complex community that influence human physiology, metabolism, nutrition and immune function, with disruption to the gut microbiota linked with gastrointestinal conditions such as inflammatory bowel disease, obesity and cancer [15].

Only a few bacterial species (e.g., *Escherichia coli*, *Bifidobacterium* sp., *Lactobacillus* sp., *Bacteroides* sp., *Eubacterium* sp.) catalyzing the metabolism of polyphenols have to date been identified [16,17,18,19]. However, they do not seem to be ubiquitous but reflect the interpersonal differences in the gut microbial community. Anthocyanins are subjected to metabolism by microbiota, and they and/or their metabolites may also modulate growth of specific bacteria from the microbiota [3]. Several data from experimental models and human subjects support the fact that changing the gut microbiota by means of diet may contribute along with several other parameters to the development of metabolic diseases associated with obesity [3]. For example, in vitro incubation of malvidin-3-glucoside with fecal slurry enhanced the growth of total bacteria, including *Bifidobaterium* spp. and *Lactobacillus* spp., with no effect observed on *Bacteroides* spp. growth [20]. Gallic acid, one of the microbial anthocyanin metabolites, was shown to reduce a group of potentially harmful bacteria such as *Clostridium histolyticum*, without negative effect on beneficial bacteria [16]. *Bifidobacterium* spp. increased in both human intervention studies and in wild-type mice supplemented with anthocyanins [21,22]. Major groups of intestinal bacteria possess β-glucosidase activity, including *Bifidobacterium* spp. and *Lactobacillus* spp. These bacterial groups are associated with beneficial effects in the intestine, including the antimicrobial effect of pathogenic microorganisms by production of short-chain fatty acids, as well as by competition for growth substrate and adhesion sites.

Still unanswered is the question of whether the observed effect in the pilot study by Kropat et al. [13] persists over a longer period of consumption and whether the results of the enriched bilberry extract are reproducible with a consumer-relevant anthocyanin-rich fruit beverage. Thus, we investigated the impact of an anthocyanin-rich fruit beverage on gut microbiota composition and oxidative biomarkers after long-term consumption in a nine-week randomized, placebo-controlled parallel intervention study with 57 healthy male volunteers.

## 2. Materials and Methods

### 2.1. Characteristics of Study Beverages

Both study beverages, anthocyanin-rich fruit juice and placebo drink, were provided by Eckes-Granini GmbH (Niederolm, Germany). The characterization and composition of the two beverages were reported in detail in [23]. The total anthocyanin content of red fruit juice was 274 mg/L, comprising malvidin-3-glucoside (mal-3-glc, 33%) followed by cyanidin-3-galactoside (cy-3-gal, 14.3%), peonidin-3-glucoside (peo-3-glc, 11.6%), petunidin-3-glucoside (pet-3-glc, 10.3%), delphinidin-3-glucoside (del-3-glc, 7.7%), cyanidin-3-arabinoside (cy-3-ara, 6.8%), cyanidin-3-glucoside (cy-3-glc, 6.4%), delphinidin-3-arabinoside (del-3-ara, 3.8%), malvidin-3-galactoside (mal-3-gal, 2.5%), petunidin-3-galactoside (pet-3-gal, 2%) and delphinidin3-galactoside, (del-3-gal, 1.6%).

### 2.2. Study Design

The nine-week human intervention study (one-week wash-out period and eight-week intervention period) had a prospective, randomized, placebo-controlled parallel design. The study was approved by the local ethics committee of Rhineland–Palatine, Mainz, Germany (no. 837.013.14 (9252-F); 28 February 2014), and started with the wash-out period 19 January 2015 and ended 31 March 2015.

### 2.3. Participants

Healthy male volunteers (*n* = 62, body mass index (BMI) = 19–25, age = 20–50) fulfilling the inclusion criteria (healthy non-smokers, BMI < 25, no practice of excessive sports, no intake of pharmaceutical drugs or food supplements during the study period) gave their informed written consent prior the intervention trial. Sixty-two participants were randomly divided into two groups (placebo group, *n* = 31 and juice group, *n* = 31). Five volunteers dropped out, with 57 participants (*n* = 30 in the juice group and *n* = 27 in the placebo group) completing the intervention study (for details [23]).

After a one-week wash-out period (no food rich in polyphenols), the volunteers consumed 750 mL (bolus) of anthocyanin-rich fruit juice or placebo drink on the first day of the study. For the following 55 days, the volunteers consumed 750 mL daily of the beverage assigned to their group in three equal portions. Blood samples were collected from all subjects on an empty stomach immediately before (0 h), as well as at 4, 8 and 24 h after bolus consumption (day 1). During the rest of the study period, blood samples were collected from subjects after 1, 4 and 8 weeks. For compliance within the study requirements, spot urine samples (50 mL) of all volunteers were collected after 1-, 4- and 8-week intervention time and the anthocyanin content quantitated. Anthropometric measurements were taken on an empty bladder each time venous blood samples were collected. During the study, the nutrient intake of volunteers was evaluated based on seven-day dietary records [23]. At the beginning and the end of the intervention study, feces samples from each participant were collected for gut microbiota analysis.

### 2.4. Isolation of Lymphocytes from Whole Blood

Lymphocytes were isolated from freshly collected human blood anticoagulated with ethylenediaminetetraacetic acid (EDTA). Blood (7 mL) was layered on 7 mL of Histopaque 1077 (Sigma-Aldrich, St. Louis, MO, USA) and centrifuged at 400x g for 25 min at room temperature. The lymphocytes were collected from the layer between the plasma and Histopaque 1077 phases, transferred into 10 mL of Roswell Park Memorial Institute (RPMI) 1640 medium (Gibco, Life technologies, Carlsbad, California, USA) supplemented with 10% fetal calf serum (FCS) and 1% penicillin/streptomycin (10,000 units penicillin and 10 mg streptomycin/mL), and centrifuged for 10 min. Next, the pellet was resolved in 6 mL of 10% FCS medium and repeatedly centrifuged for 10 min (250× *g*). Finally, cells were transferred into 1 mL of RNAlater stabilization reagent (Qiagen GMBH, Hilden, Germany) and stored at −80 °C until further experiments.

### 2.5. RNA/DNA Isolation and Quantitative Real-Time (qRT)-PCR

Total RNA for gene transcription analysis and genomic DNA (gDNA) for genotyping were isolated from lymphocytes simultaneously with the AllPrep DNA/RNA Mini Kit (Qiagen) according to the manufacturer’s instructions and stored at −80 °C until further analysis.

Complementary DNA (cDNA) for transcription analysis was obtained by reverse transcription with the QuantiTect Reverse Transcription Kit (Qiagen) following the manufacturer’s protocol. Gene-specific DNA was amplified by qRT-PCR using QuantiTect SYBRGreen Master Mix (Qiagen), cDNA (25 ng) and gene-specific primer assays (QuantiTect Primer Assays, Qiagen). The following primer assays were used: *NQO-1*: Hs_NQO1_1_SG, QT00050281; *HO-1*: Hs_HMOX1_1_SG, QT00092645; *NFE2L2*: Hs_NFE2L2_1_SG, QT00027384; *KEAP1*: Hs_KEAP1_1SG, QT00080220; *ACTB*: Hs_ACTB_1_SG, QT00095431; and *GAPDH*: Hs_GAPDH_2_SG; QT01192646. qRT-PCR was performed with the StepOnePlus system (Applied Biosystems) following the universal PCR protocol: 95 °C for 15 min, 94 °C for 15 s, 55 °C for 30 s and 72 °C for 30 s for 40 cycles. Relative transcript quantity (RQt) at a specific time point was calculated according to the comparative Ct method [24] as amplification efficiencies of the target and reference genes (*ACTB*, *GAPDH*) proved to be approximately equal.

### 2.6. Polymorphism Analysis of the NFE2L2 Promoter SNPs

Three promoter SNPs of NFE2L2 rs35352124 (-653), rs6706649 (-651), and rs6721961 (-617) were selected for genotyping analysis by pyrosequencing. To obtain the 133 bp amplicon of the region encompassing the three Nrf2 gene SNPs, 40 ng of gDNA was amplified with 4 µL of GoTaq ^®^ Flexi buffer (Promega, Fitchburg, WI, USA), 2.5 mM MgCl2, 0.2 mM deoxynucleosidtriphosphates (dNTPs), 0.4 µL of each primer (biotinylated forward primer 5′-TGCCTAGGGGAGATGTGG-3′ and reverse primer 5′-ACCTAAAGGGGCTTCTCCGTT-3′) and 0.25 units of GoTaq ^®^ Flexi DNA polymerase (Promega) to a total reaction volume of 20 µL. Amplification was performed in a VeritiTM 96-well Thermal Cycler (Applied Biosystems, Forster City, California, USA) with an initial step of 95 °C for 3 min, followed by 35 cycles of 95 °C for 30 s, 54 °C for 30 s and 72 °C for 1 min and a final extension of 72 °C for 7 min. The PCR product was verified by gel electrophoresis (2% agarose) and subsequently used for pyrosequencing with PyroMark ^®^ Gold reagents (Qiagen) according to the manufacturer’s instruction. Briefly, 10 µL of the PCR product was added to 2 µL Streptavidin Mag Sepharose beads (GE, Healthcare, Chicago, Illinois, USA) and loaded into the specific 48-well disk of the Qseq pyrosequencer (Bio Molecular Systems prototype of the Qiagen PyroMark Q48 Autoprep). After denaturation and capture of the biotinylated template strand the sequencing primer (5′-CGTGGGAGTTCAGAGG-3′) was annealed from which base incorporation was quantitated by pyrosequencing and genotypes were assigned to the sample pyrograms by QSeq software version 2.1.3 (Bio Molecular Systems). For a subset of samples, Sanger sequencing was used to confirm genotyping results; the primers 5′-GACCACTCTCCGACCTAAAGG-3′ and 5′-CGAGATAAAGAGTTGTTTGCGAA-3′ were used to amplify a 424 bp PCR product and sequenced using forward and reverse primers with a BigDye ™ Terminator v3.1 Cycle sequencing kit (ThermoFisher Scientific, Waltham, MA, USA) and capillary electrophoresis on an Applied Biosystems 3500 genetic analyzer.

### 2.7. Fecal Sample Collection and DNA Isolation

Fecal samples were collected by participants either in the morning or previous evening prior delivery in cool boxes. Samples were then aliquoted and snap-frozen in liquid nitrogen to be stored at −80 °C until analysis. DNA was extracted using a phenol-chloroform protocol. Briefly, approximately 500 µL of fecal material was disrupted using bead-beating in the presence of phenol-chloroform isoamyl alcohol (25:24:1; pH 8) and 500 µL of cetyltrimethyl ammonium bromide (CTAB) extraction buffer. Samples were centrifuged at 18,000 rcf for 5 min at 4 °C and the aqueous phase was transferred to a new sample tube. DNA was precipitated with 0.1 volume of 3 M Na-acetate and 0.6 volume of ice-cold isopropanol at room temperature for 2 h. DNA was re-suspended in 20 µL TE buffer (10 mM Tris-HCl, 0.1 mM EDTA, pH 8).

### 2.8. 16S rRNA Gene-Targeted PCR and Sequence Pre-Processing

Extracted DNA samples were subjected to two-step PCR amplification of 16 S rRNA gene using head-primers targeting all bacteria [forward primer S-D-bact-0341-b-S-17 (5′-CCTACGGGNGGCWGCAG-3′) and reverse primer S-D-bact- 0785-a-A-21 (5′-GACTACHVGGGTATCTAATCC-3′), head adaptors (5′-GCTATGCGCGAGCTGC-3′)] and 8 nucleotide-specific barcodes according to [25]. The PCR program of step 1 was: 25 cycles of 95 °C for 3 min, 95 °C for 30 s, 55 °C for 30 s, and 72 °C for 1 min, with a final step of 72 °C for 7 min. From the resulting product 2 µL were added to 18 µL of a second master mix containing individual barcoded primers for each sample. The PCR program of step 2 was: 5 cycles of 95 °C for 3 min, 95 °C for 30 s, 52 °C for 30 s, and 72 °C for 1 min, with a final step of 72 °C for 7 min. Samples were then quantified using PicoGreen (ThermoFisher scientific, Invitrogen, Vienna, Austria) pooled in an equimolar mixture and sequenced using Illumina MiSeq technology at Microsynth AG.

Sequence data were sorted into libraries, using the 8 nucleotide sample-specific barcode and primer using a custom-made house script, quality filtered according to the Earth Microbiome Project guidelines and paired-end reads were concatenated [26]. Reads were then clustered into species-level operational taxonomic units (OTUs) of 97% sequence identity, checked for chimeras using USEARCH, and taxonomically classified using the Ribosomal Database Project naïve Bayesian classifier [27]. To avoid biases related to uneven library depth, sequencing libraries were subsampled to a number of reads smaller than the smallest library (2500 reads). The sequence data were submitted to the NCBI database under bioproject number: PRJNA491813.

### 2.9. Statistical Analysis

Statistical analysis of gene transcription and genotyping results was performed using the Kruskal–Wallis test (non-parametric). A *p*-value ≤ 0.05 was considered statistically significant. The data of qRT-PCR performed in triplicate are presented as BOX-diagrams. Values are the means ± SD normalized to the mean of *ACTB* and *GAPDH* transcript levels and are presented as relative transcription (RQ), compared to transcript levels at time point 0 h, which is set to 1 (baseline).

Further statistical analysis was performed using the statistical software R version 3.5.1 [28]. The statistical significance of factors associated with microbiota composition was determined using non-parametric multivariate analysis of variance (PerMANOVA) and significant clustering of groups was evaluated with analysis of similarities (ANOSIM). Ordination analysis was performed using redundancy analysis (RDA) in the vegan package [29]. Alpha and beta diversity metrics were also calculated in the vegan package. Indicator species analysis was used to identify taxa and OTUs that are enriched under certain conditions (indicspecies package: [30]). The correlations between gut microbiota and cohort characteristics were performed using Pearson and Spearman correlation coefficients.

Phylogenetic Investigation of Communities by Reconstruction of Unobserved States (PICRUSt) 1.0.0 [31,32] was applied to predict metagenome function from the 16 S rRNA gene data; Bray–Curtis distances were used to determine similarity of samples based on metagenomic composition. Differences in the taxa and predicted molecular functions were analyzed by the linear discriminant analysis (LDA) effect size (LEfSe) [33] with default settings (Alpha value for the factorial Kruskal–Wallis test among classes = 0.05; Threshold on the logarithmic LDA score for discriminative features = 2.0).

Variables were expressed as mean ± standard deviation (SD), and for multiple comparisons, *p*-values were adjusted with the False Discovery Rate method (FDR). A *p*-value ≤ 0.05 was considered statistically significant.

## 3. Results

### 3.1. Modulation of Nrf2-Regulated Genes

**Figure 1:**
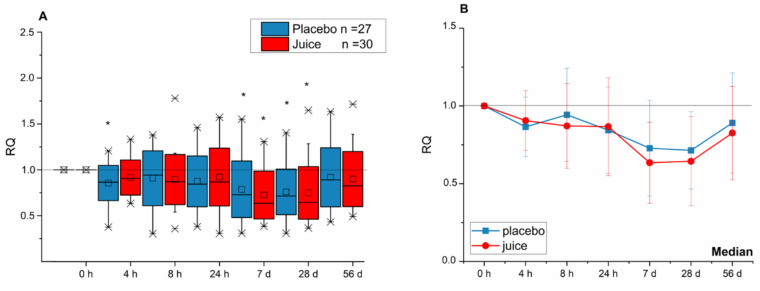
Modulation of *NEF2L2* transcript levels after intake of anthocyanin-rich multi-fruit juice (juice, *n* = 30) or placebo beverage (placebo, *n* = 27) over time of intervention. (**A**) The transcript levels are normalized to the mean of transcript levels of *ACTB* and *GAPDH* and set in relation to the control (transcript levels at time point 0 h; relative transcription (RQ)). (**B**) Line graphs represent the median values of relative transcript levels (RQ) ± SD. Significance obtained when compared to the control is calculated by Kruskal–Wallis test with * = *p* < 0.05.

Transcript levels of Nrf2/ARE-dependent genes *HO-1* and *NQO-1*, as well as *NFE2L2* (Nrf2) itself, were clearly affected in lymphocytes of the study participants after consuming the anthocyanin-rich multi-fruit juice. Effects were evident after a short time (up to 24 h) as well as over a long period (up to 56 d) intake. The transcript level of *NFE2L2* was significantly lower after 4 h and at days 7 and 28 when compared to the level at the 0 h time point (Figure 1A). At day 56, at the end of the intervention, the reduction in *NFE2L2* transcript level was still evident; however, statistical significance was not achieved. The line graphics (Figure 1B) represent the median values of relative transcript levels. Interestingly, both the placebo and juice groups showed a similar U-shape curve in the effect–time–sequence plot. No statistically significant difference between placebo and the juice group was evident.

**Figure 2:**
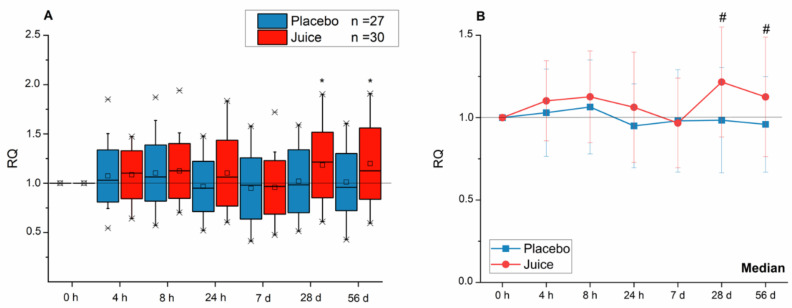
Modulation of *HO-1* gene transcription after 4-, 8-, 24-h and 7-, 28-, 56-day intake of anthocyanin-rich multi-fruit juice (juice, *n* = 30) or placebo (placebo, *n* = 27). (**A**) The relative transcript levels were normalized to the means of *ACTB* and *GAPDH* and the negative control (transcript levels at time point 0 h; RQ). (**B**) Line graphs represent the median values of relative transcript levels (RQ) ± SD. Significance compared to the control is calculated by Kruskal–Wallis test with * = *p* < 0.05; significances relative to placebo group with # = *p* <0.05.

With respect to *HO-1* transcript levels, the placebo and juice groups showed comparable effects up to seven days of beverage consumption (Figure 2). However, after 28 days, *HO-1* mRNA in peripheral blood lymphocytes (PBLs) of the intervention group was significantly elevated in comparison to the placebo group. This significant effect persisted until the end of the intervention (56 d).

**Figure 3:**
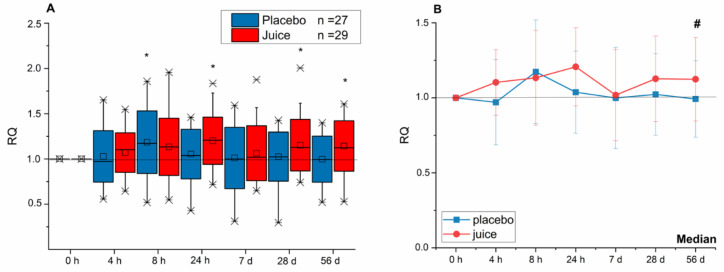
Modulation of *NQO-1* gene transcription after 4-, 8-, 24-h and 7-, 28-, 56-day intake of red multi-fruit juice (juice, *n* = 29) or placebo (placebo, *n* = 27). (**A**) The relative transcript levels were normalized to the means of *ACTB* and *GAPDH* and the negative control (transcript levels at time point 0 h; RQ). (**B**) Line graphs represent the median values of relative transcript levels (RQ) ± SD. Significance compared to the control is calculated by Kruskal–Wallis test with * = *p* < 0.05; significances relative to placebo group with # = *p* < 0.05.

In addition, the transcript level of *NQO-1* in the juice group was found to continuously increase until reaching a significant level 24 h after juice consumption (Figure 3). In the placebo group, only a significant increase of the *NQO-1* transcript level after 8 h was observed, at all other timepoints the gene transcription remained at base levels. At the end of the intervention period (56 d), the *NQO-1* gene transcription in PBLs of the juice group was significantly increased in comparison to placebo group.

### 3.2. Impact of NFE2L2-SNPs on the Response to Fruit Juice Consumption

**Figure 4:**
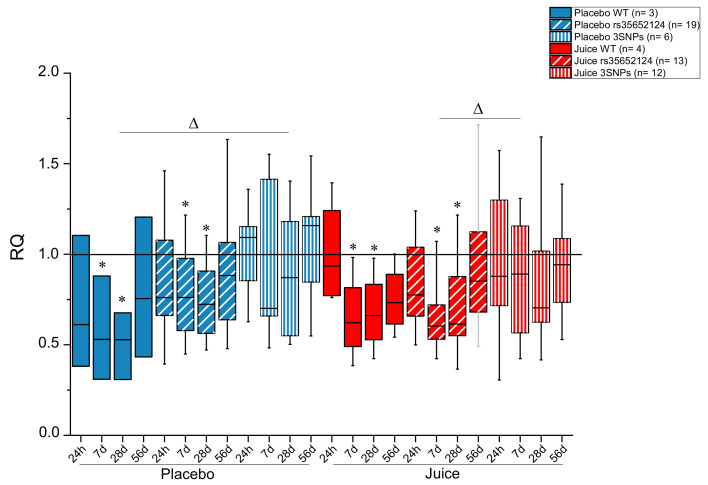
Impact of the consumption of anthocyanin-rich multi-fruit juice (Juice) and placebo beverage (Placebo) on *NFE2L2* transcript levels in peripheral blood lymphocytes (PBLs) of participants carrying different single nucleotide polymorphisms (SNPs) in the *NFE2L2* promotor. *NFE2L2* transcript levels were normalized to *ACTB* and *GAPDH* levels and related to the control (levels at 0 h; RQ ± SD). Significance compared to the control is calculated by Kruskal–Wallis test with * = *p* < 0.05; significance relative to different subgroups with Δ = *p* < 0.05. Abbreviations: WT = wild-type (without SNP); SNP rs35352124; 3SNP = Nrf2 promoter SNPs rs35352124, rs6706649 and rs6721961.

As no significant difference was observed between the placebo and juice groups with respect to Nrf2 transcription (Figure 1) the question arose whether SNPs in the promotor region might play a role in the response to fruit juice, masking the effect of responders/non-responders. The *NFE2L2*-SNPs rs35652124, rs6706649 and rs6721961, all located in the promotor region, were previously described to affect the activity of the Nrf2/ARE-system [12,33,34]. Hence, the Nrf2 genotype of the study participants was determined focusing on these three SNPs. For analysis, the two groups (placebo and juice group) were divided into three subgroups according to genotype: the wild-type (WT) group with none of the three identified promotor SNPs, the group with only the rs35652124 minor allele SNP (1SNP) and the group possessing all three minor allele SNPs (3SNP). Of note, only 7/57 probands (placebo: 3, juice: 4) were identified as wild-type carriers. Due to the unexpected small group size, any associations between genotype and biological response have to be viewed as preliminary. Furthermore, the genotypes of the participants carrying the 3SNP haplotype were not evenly distributed between both intervention groups with six participants in the placebo group and 12 participants in the juice-receiving group. Statistical analysis indicated that carriers of the wild-type allele and the SNP rs35652124 within the placebo group had significantly lower *NFE2L2* transcript levels after 7 and 28 days as compared to day zero, the beginning of the intervention (Figure 4). At day 56 transcript levels of *NFE2L2* returned to levels of day zero. Interestingly, all subgroups of the placebo group displayed a U-shape curve as observed before for the median analysis of *NFE2L2* transcript levels (Figure 1B). Comparison of the different subgroups within the placebo group suggests an increase of *NFE2L2* transcripts when more minor SNP alleles are present, with the 3SNP haplotypes group showing the highest transcript level over the whole intervention period (56 days). On day 28 a statistically significant difference in *NFE2L2* transcript levels of the placebo group was reached between WT and 3SNP (Figure 4).

In the juice group, a similar effect was observed. The WT group and the group possessing SNP rs35652124 had significantly lower levels of *NFE2L2* transcripts at days 7 and 28 of the intervention period as compared to day zero. In the 3SNP group, no significant change of *NFE2L2* mRNA levels was observed. After seven days of juice consumption *NFE2L2* transcription was found to be significantly higher in the 3SNP group compared to the 1SNP group (rs35652124, Figure 4). Taken together, the results indicated in the placebo group higher *NFE2L2* transcript levels when more SNPs were present. In the juice group, this trend was still evident, however, this was not shown to be statistically significant. Overall, consumption of fruit juice did not alter the transcript levels of *NFE2L2*, with statistical significance in the different genotype subgroups in comparison to placebo beverage intake (Figure 4).

**Figure 5:**
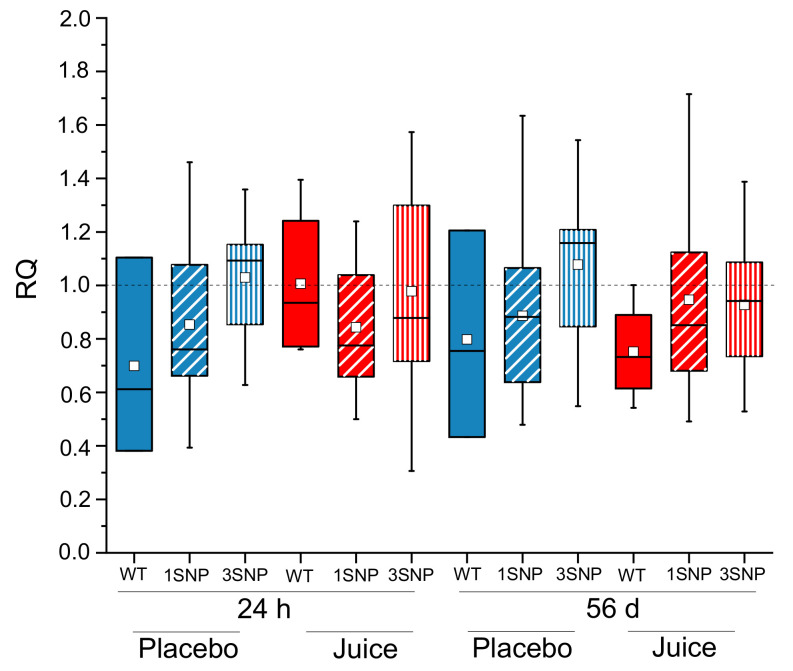
Modulation of the *NFE2L2* transcript level in PBLs after short-term (24 h) and long-term (56 d) intake of anthocyanin-rich multi-fruit juice (Juice in red) or placebo beverage (Placebo in blue) in correlation to *NFE2L2* promoter single nucleotide polymorphisms (SNPs). *NFE2L2* transcript levels were normalized to *ACTB* and *GAPDH* and related to the control (levels at 0 h). Abbreviations: h: hours, d: day; WT = wild-type (without SNP); 1SNP = Nrf2 promoter SNP rs35352124; 3SNP = Nrf2 promoter SNPs rs35352124, rs6706649 and rs6721961.

Comparison of short- (24 h) and long-term (56 days) consumption between the placebo and juice groups revealed more clearly these effects. In the placebo group increased *NFE2L2* transcript levels were observed after 1 and 56 days, specifically in 3SNPs samples (Figure 5). This picture changed after fruit juice consumption. The *NFE2L2* transcript level decreased or rather did not change after short-term consumption in samples where more SNPs were present. After long-term juice consumption of 56 days a minor increase in the transcript level supported the hypothesis that more SNPs in the promotor region are associated with higher *NFE2L2* transcript levels. However, when considering the small group sizes of the different SNP carriers, this data can only be interpreted as suggestive at this stage with respect to the impact of *NFE2L2*-SNPs investigated on the antioxidant response.

### 3.3. The Gut Microbiome Composition

**Figure 6:**
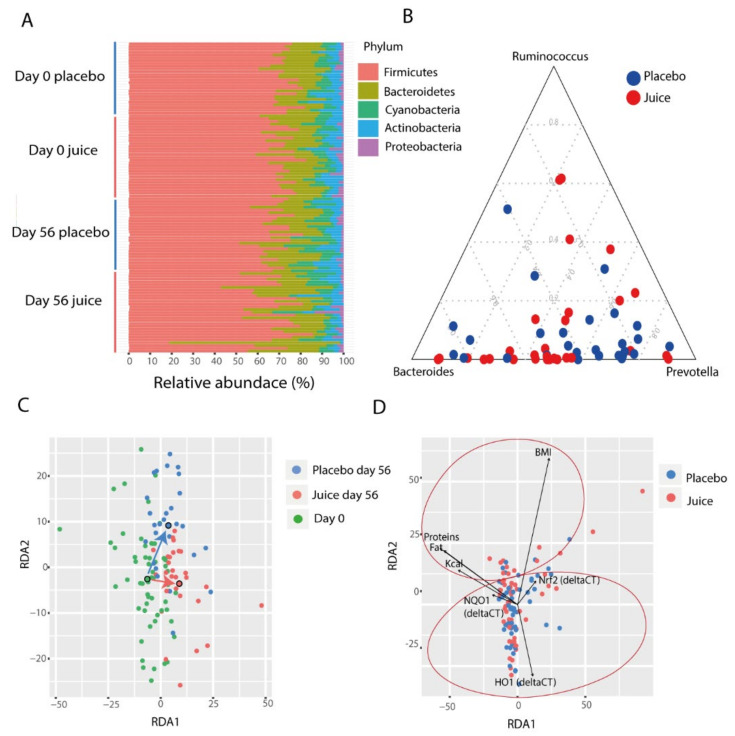
(**A**) Phylum-level composition of the gut microbiota divided by groups (juice, placebo) and time points (day 0, day 56). (**B**) Abundance of major enterotype genera at day 0. (**C**) Redundancy analysis (RDA) of the gut microbiota shows a grouping of samples. Each point represents a sample and the colors represent the groups (placebo day 56, juice day 56, day 0). The larger circles show the centroids for each group. (**D**) The significant parameters correlated with the gut microbiota are represented in the redundancy analysis plot. Significant correlation is present with nutritional parameters (top of the plot) and Nrf2 gene, *NQO1*, and *HO-1* transcript levels (bottom of the plot).

The microbiota composition of study participants was found to be typical for healthy individuals, with the community dominated by the phyla *Firmicutes* and *Bacteroidetes*, and minor phyla including *Proteobacteria*, *Actinobacteria*, and *Cyanobacteria* (Figure 6A). It has been suggested that human gut microbiota can be classified into three distinct types, called enterotypes, characterized by abundance of the genera *Bacteroides*, *Ruminococcus*, and *Prevotella* (Figure 6B). We therefore evaluated whether the abundances of these genera were different between groups or affected by the intervention. We observed that at day 0 there was no significant difference in the abundance of these groups between juice and placebo group (*p* = 0.682) (Figure 6A). There was also no significant difference at day 56 (*p* = 0.366), and the juice consumption and time did not affect these abundances (*p* = 0.19, *p* = 0.44), suggesting that the enterotype status was not altered during the course of the intervention.

### 3.4. Factors Affecting Microbiota Composition

Overall, the microbial community of the study participants was significantly affected by time and day of treatment (perMANOVA, day: *p* = 0.001, time: *p* = 0.001., ANOSIM, day: *p* = 0.01, time: *p* = 0.01), indicating that the juice and placebo groups had distinct changes occurring in the microbiota (Figure 6C). In addition, the microbiota composition was significantly affected by diet, specifically with total calorie consumption, fat, protein and carbohydrates levels (perMANOVA, total calories: *p* = 0.002, protein: *p* = 0.001, fat: *p* = 0.001, carbohydrates: *p* = 0.016). In particular, energy intake (Kcal) was found to be negatively correlated with *Bacteroidales* (r = −0.3586, *p* = 0.033) and positively correlated with *Clostridiales* (r = 0.35, *p* = 0.030). Similar to total calorie consumption correlations, fat intake was also positively correlated with *Firmicutes* (r = 0.3296, *p* = 0.044), in particular with *Clostridiales* (r = 0.4005, *p* = 0.020), and negatively correlated with *Bacteroidetes* (r = −0.3157, *p* = 0.044). Protein and carbohydrate intake were identified to be positively correlated with *Peptococcaceae* (r = 0.5190, *p* = 0.0007).

The microbiota were also associated with BMI, Nrf2 genotype status and *HO-1* and *NQO-1* expression levels (perMANOVA, BMI *p* = 0.009, Nrf2 genotype: *p* = 0.004, *HO-1* expression: *p* = 0.004, and *NQO-1* expression: *p* = 0.005). The redundancy analysis highlighted a specific grouping of these parameters. The nutritional parameters grouped on the top of the RDA while Nrf2 gene, *NQO-1*, and *HO-1* expression levels were on the bottom (Figure 6D).

**Figure 7:**
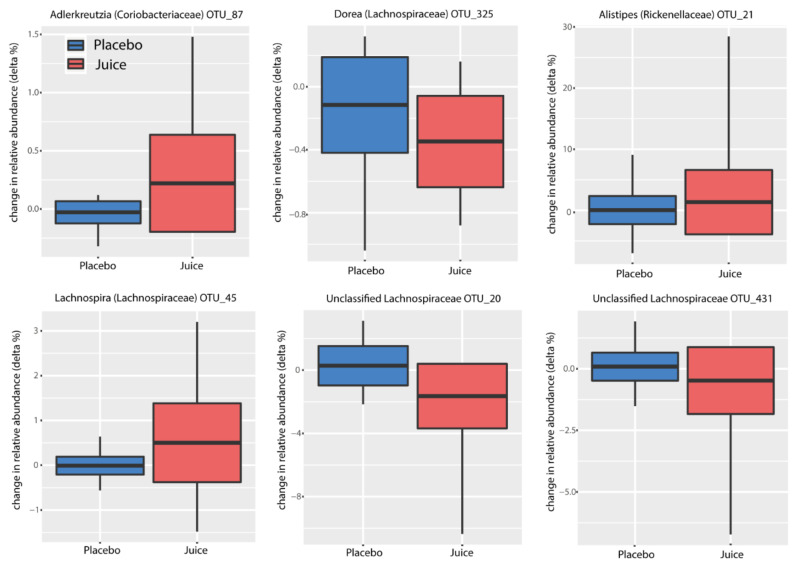
Multiple operational taxonomic units (OTUs) were identified as indicators for treatment. The plots show the change in relative abundance (change in %) for each OTU (operational taxonomic unit = 97% sequence similarity or greater).

**Figure 8:**
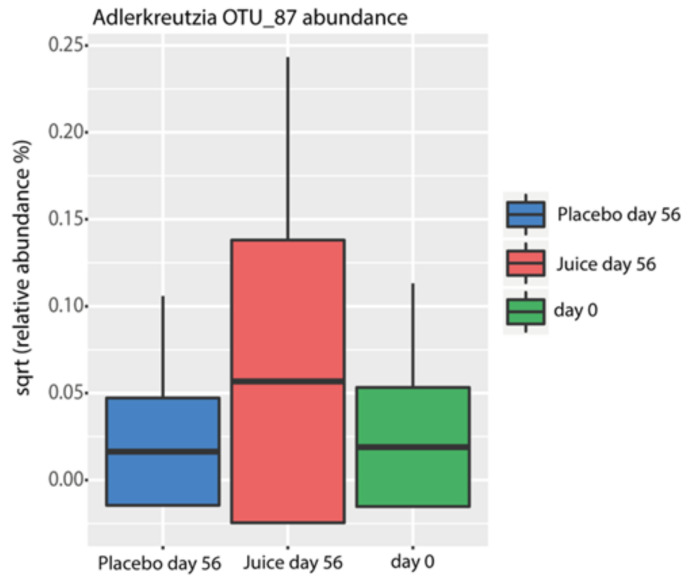
Square-root-normalized relative abundance of *Adlercreutzia* OTU 87, an indicator bacterium for treatment. (ANOVA, *p* = 0.0001, Tukey’s post hoc test, juice treatment d56–placebo d56: *p* = 0.0016, juice treatment d56–d0: *p* = 0.0002, placebo d56–d0: *p* = 0.99).

Multiple operational taxonomic units (OTUs) were identified as indicators for treatment (Figure 7). Interestingly, OTU 87, which was classified to the genus *Adlercreutzia*, was found to be enriched in the treatment group (ANOVA, *p* = 0.001) together with *Lachnospira* (OTU_45) (ANOVA, *p* = 0.0001). *Alistipes* (OTU_21) and unclassified *Lachnospiraceae* (OTU_431) showed a trend of increasing in relative abundance in the treatment group, though this did not reach statistical significance (*p* = 0.11 and *p* = 0.18, respectively). In contrast, *Dorea* (OTU_325) and unclassified *Lachnospiraceae* (OTU_20) were reduced in the treatment group (ANOVA, *p* = 0.039 and *p* = 0.024, respectively) (Figure 8).

**Figure 9:**
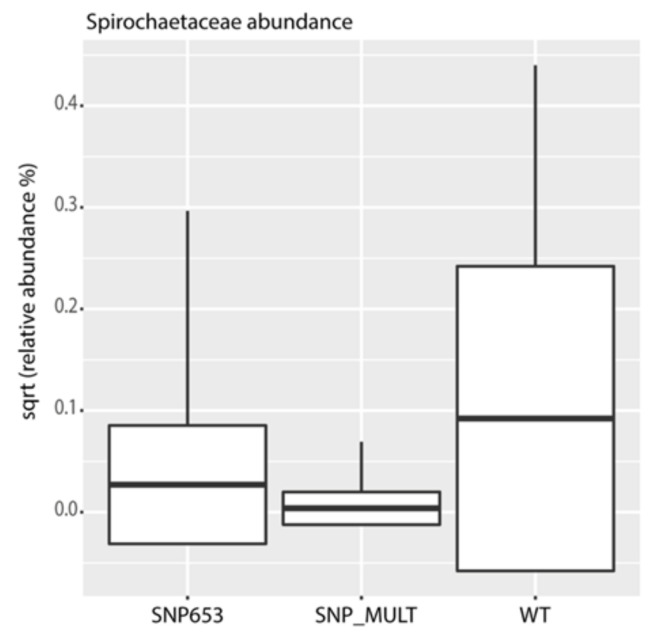
Square-root-normalized relative abundance of *Spirochaetaceae*, an indicator taxon for Nrf2 genotype. WT = wild-type (without SNP); SNP_MULT = Nrf2 promoter SNPs rs35352124, rs6706649 and rs6721961 or 3SNP. (ANOVA, *p* = 0.0006, Tukey’s post hoc test, WT-SNP653: *p* = 0.002, WT-SNP_MULT: *p* = 0.0004, SNP_MULT-SNP653: *p* = 0.59).

We also identified several microbial indicators for Nrf2 status. The most prominent feature in these indicators was an enrichment for organisms classified as *Spirochaetaceae* in participants carrying the wild-type allele as compared to individuals with the presence of multiple SNPs (Figure 9).

### 3.5. Predicted Metabolic Pathways Enriched after Fruit Juice Consumption

**Figure 10:**
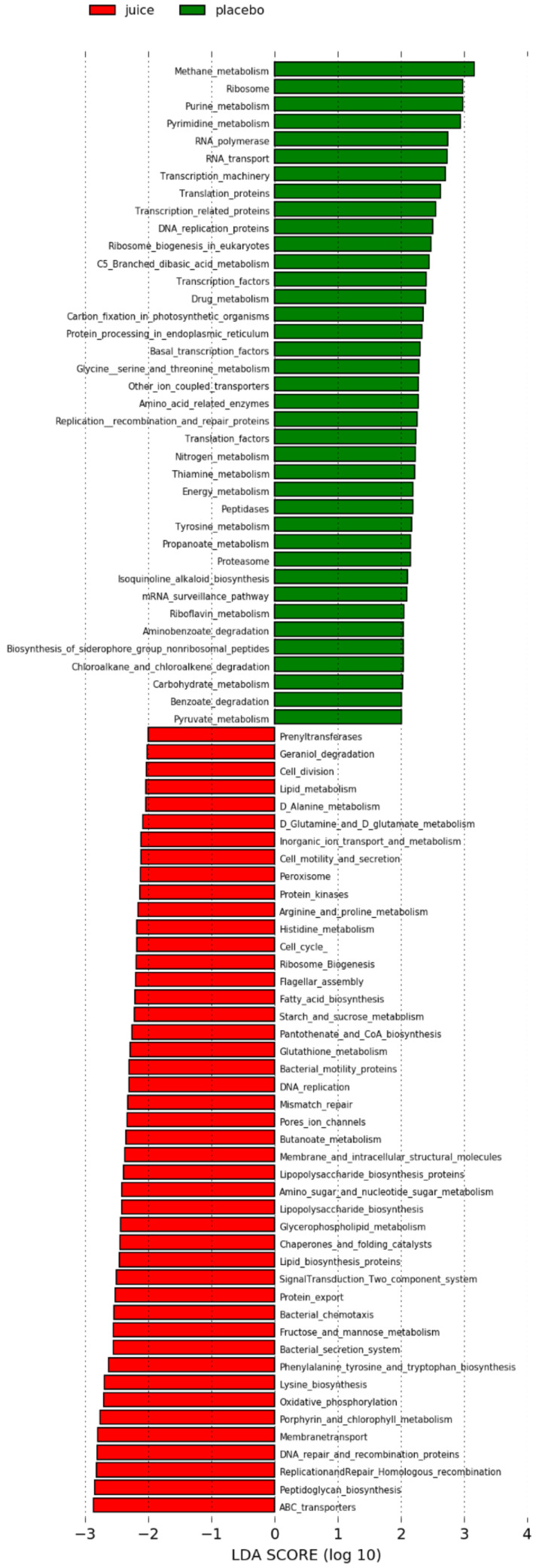
Predicted metabolic pathways enriched in the two groups of study at day 56 (juice and placebo) performed with PICRUSt Galaxy. Kyoto Encyclopedia of Genes and Genomes (KEGG) pathways differentially abundant in juice group (red) and placebo group (green) gut microbiota are shown. Alpha value for the factorial Kruskal–Wallis test between the two groups (juice and placebo) was set to 0.05 and a threshold on the logarithmic linear discriminant analysis (LDA) score for discriminative features was set to 2.0. All the significant features with a *p*-value < 0.05 are shown.

To gain insight into the molecular functions of bacterial microbiota, we used PICRUSt to predict the metagenomic contribution of the communities observed by imputing the available annotated genes within a known sequences database, the Kyoto Encyclopedia of Genes and Genomes (KEGG). PICRUSt analysis suggested significant differences (Figure 10) in several metabolic pathways in the study cohort. Significant differences were found at day 56 when comparing the placebo and juice groups, with no significant differences detected at day 0. At day 56, the juice group showed an enrichment in genes encoding enzymes for lipid [fatty acid biosynthesis (*p* = 0.0072), glycerophospholipid biosynthesis (*p* = 0.0034) and lipid metabolism (*p* = 0.0026)], carbohydrate [starch and sucrose metabolism (*p* = 0.0054), fructose and mannose metabolism (*p* = 0.0028) and peptidoglycan biosynthesis (*p* = 0.0029)]; and amino acid metabolism [arginine and proline metabolism (*p* = 0.0072), histidine metabolism (*p* = 0.004), lysine and alanine metabolism (*p* = 0.0026)]. Interestingly, these participants also demonstrated enrichment in pathways involved in DNA repair, including DNA replication and Mismatch repairing (*p* = 0.0025 and *p* = 0.0065, respectively). Other enriched pathways included cell motility and energy metabolism [phosphorylation (*p* = 0.0072), protein kinase (*p* = 0.0015), ABC transporters pathways (*p* = 0.0036)]. In the placebo group pathways involved in nucleotide metabolism, transcription and translation were found to be enriched at day 56 (*p* = 0.0036, *p* = 0.0031, *p* = 0.013, respectively).

## 4. Discussion

Recently, we reported that an anthocyanin-rich bilberry pomace extract modulated Nrf2/ARE-dependent gene transcription in a human pilot intervention study with healthy and ileostomy probands [13]. The effect was limited to healthy subjects, suggesting a role for colonic processes in bioactivity. Furthermore, we found a consumer-relevant red multi-fruit juice to be similarly effective in a short-term (24 h) pilot intervention trial [34]. Consequently, we conducted a long-term intervention study with 57 healthy male participants focusing on the impact of anthocyanin-rich fruit juice consumption on Nrf2-signaling and gut microbiota.

*NFE2L2* transcript levels were significantly decreased in placebo and juice groups after 4 h, 7 d and 28 d. However, at the end of the intervention (56 d), *NFE2L2* transcription returned to basal levels. These findings are in line with the short-term effect of bilberry extract described by Kropat et al. [13]. In the study of Kropat et al. a significant decrease of *NFE2L2* mRNA levels was detected after consumption of a bilberry pomace extract and was described as a U-shaped time-curve. [13]. In the present study also upon longer intervention a U-shaped response curve was observed but without induction of the *NFE2L2* transcription above basic level at intervention start. No statistically significant difference was identified between placebo and juice groups. Vitamin C, consumed either with the juice or the placebo beverage, might be speculated to play a role for the apparent transient decrease in *NFE2L2* transcription in both groups. However, Nrf2-dependent *NQO-1* transcript levels were significantly increased in the juice group at the completion of the intervention (56 d) in comparison to the placebo group. The increased *NQO-1* is in accordance with the short-term effects of bilberry extract in the study of Kropat et al. [13]. Moreover, *HO-1* was significantly enhanced after 28 days of juice consumption and maintained this increased level until the end of the intervention. *NQO-1* and *HO-1* are both enzymatic regulators of antioxidant capacity and have been found to be associated with different diseases, including cancer and Alzheimer’s disease [35,36,37].

As all subjects were instructed to abstain from polyphenol-rich food sources [23]; it might be speculated that under these special dietary conditions Nrf2 activity was already affected, potentially contributing to the reduced *NFE2L2* transcript level observed in both groups. In addition, both beverages contained relatively high amounts of vitamin C (>500 mg/mL), which modulates Nrf2 activity [38,39]. As such, the anthocyanin/other antioxidant content of the juice might not have been high enough to significantly induce *NFE2L2* transcription or the vitamin C levels masked the effect. However, the consumption of fruit juice potently increased *HO-1* and *NQO-1* mRNA levels, suggesting a potential impact on the Nrf2-pathway in PBLs and suggesting a systemic effect.

We additionally followed a nutrigenomic approach by investigating three different *NFE2L2*-promotor SNPs already known to be physiological relevant. Allele frequencies of rs6706649 and rs35652124 SNPs were in line with the current literature and within the normal reported HapMap frequency in Caucasians [40,41]; 19% (11/57) of the study participants carried the SNP rs6706649 minor allele and 56% (32/57) had SNP rs35652124. A total of 14% of participants carried the SNP rs6721961 minor allele (8/57), which is a lower incidence than reported by Marzec et al. and Hassmann et al. [12,33,34], although similar to the gnomAD frequency of 12% for non-Finnish Europeans. The preliminary data suggest that carriers of increasing numbers of SNPs in the placebo group displayed elevated *NFE2L2* transcript levels in comparison to WT carriers. After fruit juice consumption, this effect was no longer evident (Figure 5), pointing to a potential role of genotype in the response to juice consumption. Our findings are in accordance with effects observed for coffee beverage by Boettler et al. and Hassmann et al. [12,42]. They also reported for carriers of the SNP rs6706649 and rs6721961, an elevated basal level of Nrf2 gene transcripts in the placebo group. Furthermore, they observed a genotype-dependent induction of Nrf2 transcription by coffee consumption. However, in this study, consumption of anthocyanin-rich juice reduced Nrf2 gene transcription or had no effect (Figure 5). These data demonstrate the relevance of specific Nrf2 genotypes for the regulation of the antioxidant Nrf2 signaling and the response to anthocyanin-rich beverages.

Additionally, we could show for the first time that the gut microbiota are influenced by the Nrf2 genotype. The microbiota composition of study participants was found to be typical of healthy individuals [43,44]. The juice intake had a small but significant impact on the composition of the microbiota, specifically demonstrating this effect when examined by day and treatment (placebo or juice). We observed an enrichment of organisms classified as *Spirochaetaceae* in individuals carrying the WT allele in contrast to multiple SNPs carriers in the cohort. We hypothesize that Nrf2 polymorphisms might have an impact on mucosal barrier function and redox balance, influencing the ability of *Spirochaetaceae* to colonize the gut. These findings seem to identify *Spirochaetaceae* as an indicator taxon of the Nrf2 genotype. Interestingly, the colonization of *Adlercreutzia* (OTU 87) was enriched in the juice group. *Adlercreutzia* is known to be involved in steroid metabolism and can metabolize daidzein to equol, suggesting that enrichment of this group may provide a health benefit for individuals consuming a diet including soy products [45]. In addition, no potentially harmful taxa were identified to be enriched by juice consumption. These findings indicate that the juice and placebo groups produced distinct changes in microbiota. In agreement with these observations, functional inference analysis by PICRUSt showed that the juice group at day 56 was enriched for genes encoding enzymes mostly involved in the metabolism of carbohydrates, lipids and amino acid and in pathways involved in DNA repair.

Moreover, in our study we found that calories and macronutrient intake influenced the gut microbiota composition. Certain members of *Firmicutes* were negatively correlated with *Bacteroidetes*, which were positively correlated with calories and macronutrient intake, in line with previous reports [46,47]. As yet, no systematic review has assessed the role of xenobiotic biotransformation in the colon, in particular, with regard to anthocyanins. However, these data show that it is necessary to take the gut microbiome composition into account for analyzing the impact of anthocyanin-rich juices on antioxidative biomarkers of human health. This is also supported by several animal and human intervention studies [48,49]. In addition, in the study by Kropat et al., participants with an ileostomy (lacking a colon) had a different Nrf2-dependent gene transcription profile when compared to healthy study participants [13]. This indicated that an intact colon is necessary for the activation and metabolism of anthocyanins.

## 5. Conclusions

Long-term consumption of an anthocyanin-rich fruit juice affects Nrf2-dependent gene transcription in association with Nrf2 genotype and gut microbiota. These results demonstrate antioxidant effects of a consumer-relevant fruit juice in vivo focusing on Nrf2, the master regulator of oxidative stress. In addition, the individual genetic variability and the composition of the gut microbiota seem to play a crucial role in the response to fruit juice consumption.

## Data Availability

Raw data will be made available upon reasonable request within the rules of protection of data privacy and the ethical approval.

## Abbreviations

ARE/EpRE	antioxidant/electrophile response elements
PBL	peripheral blood lymphocytes
Keap1	Kelch-like ECH-associated protein 1
*KEAP1*	gene name of Keap1
Nrf2	nuclear factor erythroid 2 (NFE2)-related factor 2
*NFE2L2*	gene name of Nrf2
*NQO-1*	NAD(P)H quinone oxidoreductase 1
*HO-1*	heme oxygenase 1
qRT-PCR	quantitative real-time polymerase chain reaction
RQ	relative quantity
